# MRI and Motor Outcomes in Children with Cerebral Palsy

**DOI:** 10.15844/pedneurbriefs-29-8-3

**Published:** 2015-08

**Authors:** Deborah Gaebler-Spira, Kristen McCormick

**Affiliations:** 1 Rehabilitation Institute of Chicago; and Departments of Pediatrics and Physical Medicine and Rehabilitation, Northwestern University Feinberg School of Medicine, Chicago, IL

**Keywords:** Cerebral Palsy, Diffusion Tensor Imaging, GMFM-66, Structural Connectome

## Abstract

Investigators from University of Melbourne, Monash Children's Hospital, Royal Children's Hospital & Murdoch Children's Research Institute sought to identify correlation between magnetic resonance imaging (MRI) characteristics including white matter injury (WMI) in children with cerebral palsy (CP) and severity in motor outcomes later in life, irrespective of CP subtype.

Investigators from University of Melbourne, Monash Children's Hospital, Royal Children's Hospital & Murdoch Children's Research Institute sought to identify correlation between magnetic resonance imaging (MRI) characteristics including white matter injury (WMI) in children with cerebral palsy (CP) and severity in motor outcomes later in life, irrespective of CP subtype. Their goal was to develop a severity classification for WMI that can discriminate children with different levels of functional ability. Data was collected from children seen at Melbourne Children's Campus, in a cohort population from 1999-2008. A total of 272 children were identified from the Victorian Cerebral Palsy Register with a diagnosis of CP and WMI on MRI. Their MRI findings were divided into broad patterns of injury that reflected pathogenesis & timing of injury: unilateral, asymmetrical, or symmetrical, then based on location: anterior, mid, and posterior white matter. These data were then analyzed using Chi square tests to assess the strength of association between known Gross Motor Function Classification System (GMFCS) levels and symmetrical WMI, and between motor topography groups for children with symmetrical WMI & bilateral CP. There were clear associations between extent of signal abnormality in the worse hemisphere and laterality/symmetry of WMI (p = 0.004), extent/location of WM loss & laterality/symmetry (p < 0.001), and GMFCS/motor topography and MRI laterality/symmetry (P < 0.001). Despite the strong association between MRI symmetry & motor topography, there was only 56% agreement between the two. While WMI seen on imaging correlated with physical exam findings in a large majority of patients, physical exam findings correlated with MRI findings less frequently. Overall, it seems that the best model for predicting GMFCS level was MRI laterality/symmetry, extent of WM loss, cerebellar abnormality, and thinning of the corpus callosum (p < 0.001). These findings were used by the authors to create a new WMI severity classification for children with CP that could provide valuable predictors of future function to families. [1]

COMMENTARY. This article continues the quest to link structural brain findings to functional outcome and severity of condition [2, 3]. The relationship of structure linked to function is of increasing importance as the process of targeted neuro-rehabilitation and recovery of the impact of injury to the neonatal brain is also progressing [4]. With 56% of variance or severity yet explained, the linkage is still elusive. This is the link between parent's and neurologist, what is seen on scan and what is expected in real life.

Until a unified approach to reporting MRI findings with validated outcomes in function are used, the opportunity exists to advance the link, especially with the use of the population-based registries in Australia, Scandinavia and Europe [5, 6, 7]. Population registry methodology is the most efficient means to this end. Cooperation and collaboration of registries in the United States would increase available data which could and would advance the remaining unexplained variance of function seen in this study.
