# Correction

**DOI:** 10.15844/pedneurbriefs-29-8-3-c

**Published:** 2016-01

Corrected article: Gaebler-Spira D. and McCormick K. MRI and Motor Outcomes in Children with Cerebral Palsy. Pediatric Neurology Briefs. 2015;29(8):60. http://doi.org/10.15844/pedneurbriefs-29-8-3

The Editors of *Pediatric Neurology Briefs* regret mistakes that appeared in the original publication of this paper, as the result of production errors. The source article citation was incorrect and the source article citation was inadvertently omitted from the reference list. The correct source article citation is: Reid SM, Ditchfield MR, Bracken J, Reddihough DS. Relationship between characteristics on magnetic resonance imaging and motor outcomes in children with cerebral palsy and white matter injury. Res Dev Disabil 2015 Oct-Nov;45-46:178–87. The HTML and PDF versions of this article have been changed to reflect these corrections.

